# Physician perceptions of artificial intelligence in Northern Italy healthcare: a survey of fears and expectations

**DOI:** 10.3389/frai.2025.1624789

**Published:** 2025-11-12

**Authors:** Pietro Torricelli, Cecilia Torricelli, Beatrice Bertelli, Matteo Sandi, Annarita Pecchi

**Affiliations:** 1 Division of Radiology, Department of Medical and Surgical Sciences of Children and Adults, University of Modena and Reggio Emilia, Modena, Italy; 2 Department of Economics and Finance, Catholic University of the Sacred Heart, Milan, Italy; 3 Marco Biagi Economics Department, University of Modena and Reggio Emilia, Modena, Italy

**Keywords:** artificial intelligence (AI), survey, public health, benefits, negative effects, training, employment, mood

## Abstract

**Introduction:**

Artificial Intelligence (AI) is more and more spreading but despite the clear evidence of benefits related to its implementation, many physicians worry about ethical, legal, employment and professional changes that AI is going to induce. The purpose of this paper is to assess whether and why physicians worry about AI.

**Methods:**

This study is a cross-sectional survey addressed to a group of 362 Northern Italy hospitals physicians, both specialists and residents from selected specialties were asked to fill in a 27 multiple-choice online survey submitted by e-mail. The survey aimed to evaluate their opinions and expectations about the impact of AI on clinical, employment and ethical topics. The results were evaluated by the software Stata that enabled to carry out a multivariate analysis with the evaluation of the statistical significance of the results obtained.

**Results:**

176 physicians (48%) answered the survey. The knowledge of the topic “AI” was reported as mild in 47%, poor in 30% and good in 15%; 98% of the responders believe that AI will improve medical activities, in particular by reducing medical errors. The legal problems, the worsening of the relationship with the patients and the deep changes of the medical role have been considered its most negative expected consequences. From an employment point of view, most responders believe that the AI cause the replacement of physicians by other professional figures. The most frequent sensations caused by AI are optimism (34%), worry (30%) and enthusiasm (13%), while anxiety is reported by 9% of the responders. The responders also believe that new dedicated digital technologies and new skills will be needed. Deep changes in the formation of physicians and residents are deemed to be necessary. Gender influences the response given on the effects of AI: women tend to be overall more pessimistic, predicting greater impacts on training, with a substantially negative feeling and with a lower probability of easing litigation. The responses are not correlated with the doctor’s specialty of the respondent. The region, which influences the responses on training and feelings, does not influence the response on the effect of AI on litigation. The respondents’ origins in some regions of northern Italy and the selection of some medical specialties must be considered limitations of the reported analysis.

## 1 Introduction

Artificial intelligence (AI) is the discipline of creating algorithms and systems that can perform activities which, if performed by humans, would require intelligence. These activities include learning, reasoning, planning, and the perception and interpretation of natural language. In Italy, as in the rest of the world, AI has experienced exponential growth and aroused growing interest in many sectors, ranging from academic research to industry. AI has a transversal impact on many productive areas, such as manufacturing, transport, finance and agriculture. However, the sector that will be most affected by AI, due to the multiplicity and peculiarities of its aspects, is certainly healthcare (Vidali, 2024). AI-based systems are becoming increasingly prevalent in the medical field, with the aim of analyzing and interpreting the vast amounts of data available from numerous sources today (Kelly et al., 2022). In recent years, manufacturers have focused on developing AI models that can predict disease onset, make early diagnoses and identify treatments based on the latest scientific evidence. The diagnostic imaging sector, in particular, is interested in the growth and diffusion of AI (Kelly et al., 2022; Van Leeuwen et al., 2021; Van Leeuwen et al., 2024). Despite their advanced capabilities, AI systems struggle to be integrated stably into clinical practice and care pathways, and healthcare professionals’ attitudes towards them range from extreme optimism to distrust or open hostility (Van Leeuwen et al., 2021; Van Leeuwen et al., 2024). Furthermore, this innovative technology is not without risk; authors such as Stephen Hawking have even declared that “The development of full artificial intelligence could spell the end of the human race.” Its use, therefore, must be implemented carefully and in a controlled manner, remembering that we are dealing with an environment, the medical one, where the risk is extremely high, since it involves the lives of patients. The main barriers to the full acceptance of AI include the lack of sufficient evidence of safety, reliability and effectiveness, the absence of specific regulations for hospital use, the difficulties in determining responsibilities in case of errors or malfunctions, and the ethical and privacy issues, as well as the organizational and occupational repercussions that it may have on healthcare personnel. The importance of these arguments is at the basis of the WHO consensus ethical principles for the use of AI for health, which have defined some principles to regulate the use of AI in the healthcare sector, including responsibility and accountability. Some specialist areas, particularly in the field of diagnostic imaging, look at AI as a highly disturbing agent of their activity and role, which in some cases is even considered at risk of extinction (Aung et al., 2021, Castagno and Khalifa, 2020, Petersson et al., 2022, Waymel et al., 2019, Apell and Eriksson, 2023, European Society of Radiology, 2019). There are several theoretical models on the acceptance of AI in various industrial sectors including healthcare, from the classic TAM and UTAUT to more recent models that seek to overcome its limitations and above all introduce greater explanatory power of affective variables, as reported in the review by Chen et al. (2025). The use of questionnaires or interviews about the topic of AI has already been used and reported in the literature direct to different populations of responders (Castagno and Khalifa, 2020; Petersson et al., 2022; Waymel et al., 2019; Apell and Eriksson, 2023; European Society of Radiology, 2019).

In this direction, this paper analyzes doctors’ opinions on the growing impact of AI on their profession, their level of acceptance, and the presence of emotional states such as anxiety or worry. We also considered the educational aspect, assessing opinions on the need to modify training programs to adapt to the emerging role of AI.

Some literature reviews have demonstrated that applying the TAM and UTAUT theories on technology acceptance to the healthcare context requires taking into account the organizational complexity of the healthcare system and how certain factors and moderators, such as social influence, are more important (Afonso et al., 2012; Lee et al., 2025). Based on literature, we have formulated some hypotheses, in particular we hypothesized that there may be different behavioral attitudes in the adoption and use of AI based on the age, gender, geographic region, and area of medical specialization of the respondents.

## 2 Materials and methods

The population includes a group of 362 Graduates in Medicine and Surgery or other Specialist Degrees in the Health Sector, working in the Italian Public Health System who were asked voluntarily to answer a 27 multiple-choice online survey submitted by e-mail.

The questionnaire was introduced with information regarding the purpose of the survey. The entire questionnaire is anonymous, and it is not possible to trace the identity of the respondent from the answers. The introduction to the questionnaire explicitly stated that submitting responses would be interpreted as authorization to analyze the content, which would be used only for research and non-commercial purposes. Informed consent was implied once the “submit” button was pressed. As the study does not involve vulnerable subjects and the risks of informational or psychological harm are minimal, ethical oversight by an Ethical Review Board was deemed not to be necessary (Whicher and Wu, 2015; Castagno and Khalifa, 2020).

The mailing lists were provided by the Directors of the Clinical Structures involved and all interviewees were notified of the voluntary nature of the adhesion, the guarantee of anonymity, the confidentiality of the data and their use for exclusive research purposes.

In order to intercept any “generational” differences, we submitted the survey both to specialists and to residents, the latter presumably more involved by the impact of AI.

Before developing the survey, several focus groups were held to discuss the main themes and develop the survey questions. The focus groups included the authors of the research and individuals representing the categories to whom the survey was subsequently administered. This preliminary experience allowed us to develop the questions and verify their comprehensibility.

The questionnaire was created using QUALTRICS XM Software, and was sent via email with an access link that allowed anonymous compilation, electronic return and subsequent analysis and processing of the answers.

The survey (Supplementary Figure 1) aimed to evaluate the opinions and expectations of the interviewees about the impact of AI on clinical, employment and ethical topics.

The first part of the survey (Section 1) concerns personal (year of birth, gender, place of work) or curricular data (qualification, type of specialization), while the subsequent questions (Section 2) aim to evaluate the competence of the operators with the AI and the experiences already acquired on its use in the healthcare or research field.

The largest part of the survey is Section 3 which initially explores the benefits and the negative consequences of the implementation of AI use in the different specialist areas; afterwards the survey explores the opinions relating to the possible occupational implications of AI and the opinions relating to the changes, particularly organizational and technological, that the diffusion of AI could make necessary, both in the training and in healthcare activity. The last question of the Section 3 is about the psychological status (anxiety-optimism-worry etc.) induced from the think of IA in the future working.

The opinions about the legitimacy, ethics and medico-legal aspects related to the exclusive or complementary use of AI in diagnostic-therapeutic pathways are in the final part (Section 4).

### 2.1 Data analysis

All answers were collected, analyzed and processed.

The responses to the questionnaire were subjected to statistical analysis to identify the variables that positively or negatively relate to the detected opinions and moods.

The main objective was to determine whether characteristics of respondents, such as age, gender, region of origin and, area of specialization, were correlated with greater or lesser openness to the use of AI in medicine.

To this end, after some preliminary univariate analyses, multivariate analyses were conducted using Stata software to estimate regression models where the dependent variables of interest (see A.B.C below) were regressed on factors likely to be associated with the responses (see 1–4 below). The statistical significance of the results obtained allowed to conclude on the relevance of the assumed associations.

Specifically, to understand how doctors perceive AI, regarding their future careers in light of the increasingly prominent role of AI and the need to modify training programs to improve their approach to AI and their state of mind, we selected three questions (dependent variables) from the questionnaire that the initial focus group considered crucial and transformed them into variables to be included in the univariate and multivariate statistical analyses. We considered also which factors influence doctors’ opinion. We identified in this way regressors or independent variables for econometric analysis.

The three dependent variables constructed from questionnaire were:

- Changes in the Doctor’s Educational Path: A binary variable representing the response to the question, “Do you believe that the spread of AI will lead to changes in the doctor’s educational path?” Possible answers are “Yes” or “No,” and the variable was directly created from the corresponding questionnaire responses.
- Feelings about AI and Future Career: A categorical variable representing the response to the question, “What feeling does the topic of AI generate regarding your future career?”

There were many possible answers to this question, some not too different from each other and this would have caused a dispersion of the answers that was not optimal for the multivariate analysis. Therefore, the variable Feelings is recoded equal to 1 (Negative) if the answer included “concern” and “anxiety,” equal to 2 (Indifference) if the answer included “other” and “Neutral,” equal to 3 (Positive) if the answer included “optimism” and “enthusiasm.”

**Figure 1:**
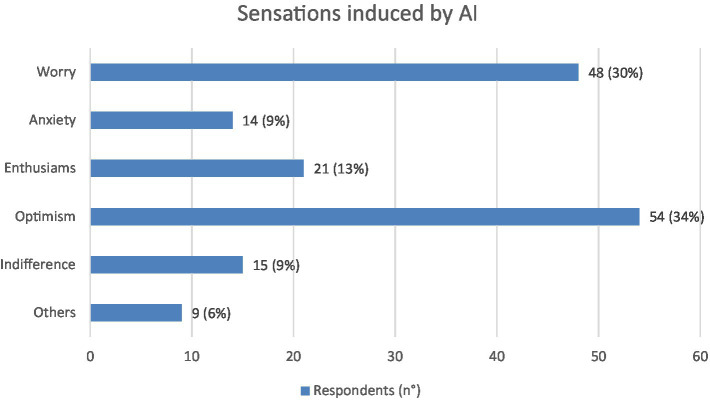
This graph reports the sensations of the responders (*N* = 161) induced by AI. Values shown next to each bar represent the number of respondents who selected that option, with the corresponding percentage in parentheses. The prevailing feelings are discordant: worry (*N* = 48) and optimism (*N* = 54).

This grouping was done primarily for numerical reasons, as some categories (especially Anxiety and Other) were underrepresented as shown in Figure 1, ensuring a more balanced and robust analysis.

- Professional Liability and AI: A binary variable representing the response to the question, “Do you believe that, in case of medical-legal disputes, a doctor’s professional liability would be reduced by the use of AI?” Possible answers are “Yes” or “No,” and the variable was directly created from the corresponding questionnaire responses.

The selection of the regressors—that is, the factors hypothesized to be related to the responses—was motivated by the aim to capture a range of personal and professional factors that could influence doctors’ attitudes toward AI.

To this end, we used all the information over respondents, i.e.:^1^

1. Year of Birth: A variable categorized into classes based on the respondent’s year of birth. It is naturally expected that age may correlate with attitudes toward new technologies such as AI. This variable tests whether age is significantly associated with the opinions about AI.
2. Gender: A binary variable distinguishing between male and female doctors. Gender may influence the perception and acceptance of new technologies. Previous studies have shown that gender differences can manifest in various ways, including approaches to technological innovation. This variable allows exploration of whether significant differences exist between men and women in attitudes toward AI.
3. Region of Origin: A categorical variable representing the geographic region where the doctor works. It can be divided into different categories corresponding to the various regions of the country. Regional differences may reflect variations in resource access, training, and exposure to emerging technologies like AI. Health policies and technological infrastructure can also vary significantly from region to region, influencing doctors’ opinions. Due to the high number of doctors practicing in Emilia-Romagna (107 respondents), a new binary variable (No ER) was later created to distinguish between doctors from Emilia-Romagna and those from other regions (ER vs. NONER).
4. Medical Specialty: A categorical variable indicating the specific medical field in which the doctor is specialized, such as Radiology, Pathology, Cardiology, etc. Different specialties may have different needs and interactions with AI technologies. This variable helps explore whether a doctor’s specialty influences their attitude toward AI. Since 113 respondents are specialists or residents in radiology, a new binary variable (Radiologist) was later created to distinguish between radiologists and doctors in different fields.

Analyzing these factors provides a deeper understanding of how different demographic and professional characteristics are related to the acceptance or rejection of AI technologies in the medical field.

Since Stata requires a numerical dataset, it was necessary to convert all verbal responses into numerical values and missing responses into points (.).

The multivariate regression analysis was performed using a Logistic Regression model for the binary dependent variables such as “educational path” and “professional liability,” while an ordered logistic regression model was used for the ordinal dependent variable “feelings” with three different possible categories (negative, positive, and indifferent).

## 3 Results

### 3.1 Data analysis

#### 3.1.1 Section 1: personal and curricular data of the responding population

Out of a total of 362 survey sent, 176 physicians responded, with a response rate of 48%, 51% males and 49% females; 48% were radiologists, both specialists and residents.

The analysis of the age groups of the interviewees highlighted that almost half n. 73 (44%) of the responses came from doctors born between 1990 and 2000 and therefore almost exclusively represented by residents or specialists in the first years of their activity, while only n. 9 (5%) respondents were born before 1960, therefore probably in the final years of their career. The remaining respondents are fairly evenly distributed between the 1980–1990 n.36 (22%), 1970–1980 n.27 (16%) and 1960–1970 n. 22 (13%) birth cohorts.

Almost all of the interviewees had a degree in Medicine and Surgery, only 2% had other types of Health Degrees. Approximately two thirds of the interviewees had a Specialization Diploma, with a large prevalence of specialization in Radiology n. 75 (48%), followed by Cardiology n. 14 (9%) and Pathological Anatomy n.10 (6%), almost equally distributed in the group of the resident: Radiology n. 38 (67%), followed by Cardiology n. 3 (4%) and Pathological Anatomy n. 2 (3%).

The most prevalent regional workplace was Emilia-Romagna: n. 107 (64%), followed by Friuli-Venezia-Giulia n. 45 (27%) and Piedmont n. 9 (5%), while the remaining shares were marginal.

A diagram showing the number of physicians invited and those who completed the survey, with respondents further classified by gender, age, educational level, medical specialty and region, is available in the Supplementary material.

#### 3.1.2 Section 2: personal competence of using AI in clinical and research activities

**Figure 2:**
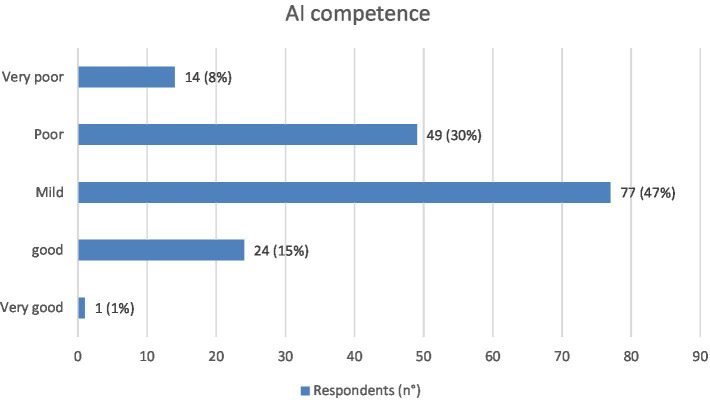
Level of AI competence reported among physician respondents (*N* = 165). Values shown next to each bar represent the number of respondents who selected that option, with the corresponding percentage in parentheses. The most frequent response was “mild”; only 1/165 believes they have very good competence.

Competence and personal familiarity with the topic of AI were defined as mild by 77 (47%) of respondents, poor by 49 (30%), good by 24 (15%), very poor by 14 (8%), while only one (1%) respondent rated them as excellent (Figure 2).

**Figure 3:**
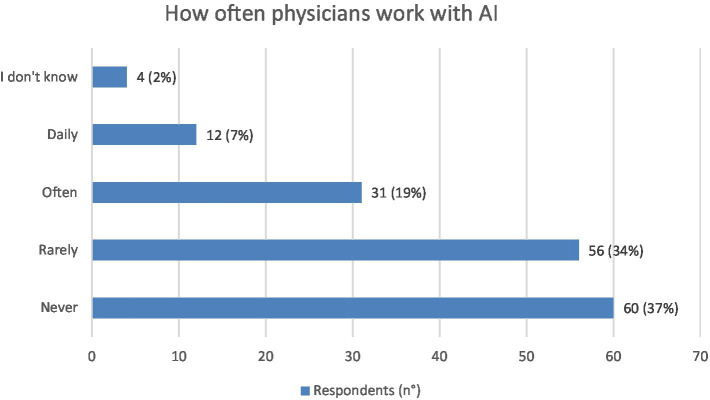
Graphic representation of how often the physician respondents (*N* = 163) work with AI. Values shown next to each bar represent the number of respondents who selected that option, with the corresponding percentage in parentheses. The most frequent responses was “never” or “rarely.” Only 4/163 respond “I do not know.”

The majority of respondents reported that they have never: 60 (37%) or rarely: 56 (34%) carried out clinical activities involving the use of AI systems, while 31 (19%) and 12 (7%) stated they have done so often or daily, respectively (Figure 3).

A clear majority of the sample also reported that they have never n. 105 (64%) or rarely n. 37 (22%) conducted research activities related to AI applications, while only 19 (12%) and 1 (1%) said they had done so often or on a daily basis, respectively.

Radiology (154: 93%), Pathology (97: 59%) and Neurology (49: 30%) were indicated as the three Medical Specialties most predictably affected by the impact of AI.

#### 3.1.3 Section 3: benefits and the negative consequences of the implementation of AI on the healthcare system and student training

**Figure 4:**
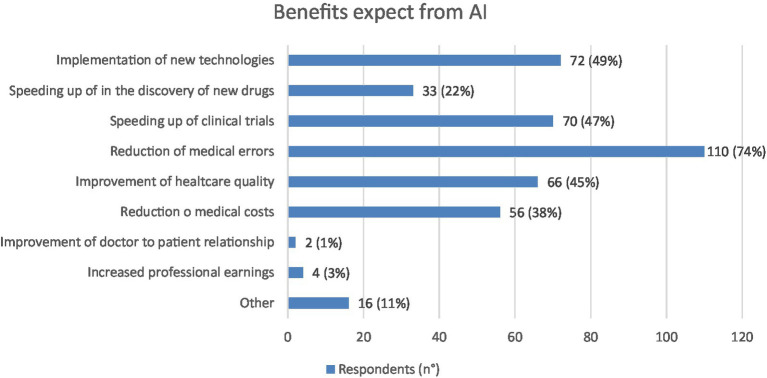
Graphic representation of the benefits expected from AI by the respondents (*N* = 148), each of whom was asked to indicate their three main perceived benefits. Values shown next to each bar represent the number of respondents who selected that option, with the corresponding percentage in parentheses. 110 physicians expect a reduction of medical errors. Not all respondents indicate three preferences.

The 98% of the sample believes that the use of AI will bring benefits in the healthcare sector and the three main expected benefits were, in order, the reduction of medical errors: 110 (74%), the implementation of new biomedical technologies: 72 (49%) and the speeding up of medical trials: 70 (47%) (Figure 4).

**Figure 5:**
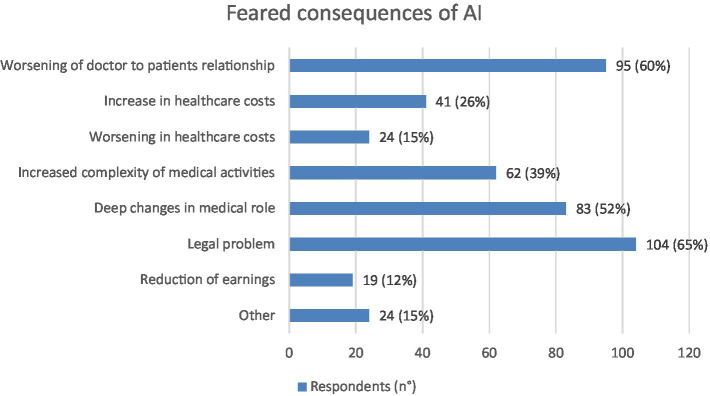
Graphic representation of the most feared consequences of AI according to respondents (*N* = 159), each of whom was asked to indicate their three main perceived negative consequences. Values shown next to each bar represent the number of respondents who selected that option, with the corresponding percentage in parentheses. Legal problems and worsening of doctor to patients relationship were the most feared consequences expected from physicians. Not all respondents indicate three preferences.

On the contrary, the three main negative effects expected from the use of AI were the medico-legal consequences: 104 (65%), the worsening of doctor to patients relationship: 95 (60%) and deep changes in medical role: 83 (52%) (Figure 5).

**Figure 6:**
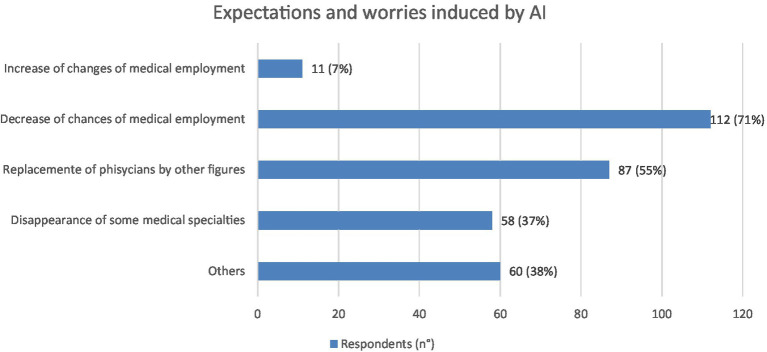
Graphic representation of expectations and worries induced by AI in physicians interviewed (N = 158), each of whom was asked to indicate their three main perceived consequences. Values shown next to each bar represent the number of respondents who selected that option, with the corresponding percentage in parentheses. The most expectations was the decrease of chances of medical employment (*N* = 112); on the other hand 11 expect an increase of chances of medical employment. Not all respondents indicate three preferences.

With regard to the impact of AI on medical employment, the main expected consequences were the decrease of chances of medical employment: 112 (71%) and the replacement of physicians by other figures 87 (55%) (Figure 6). Still in the area of employment repercussions, 65% of the sample believe that there are no specialist areas at risk of extinction as a consequence of the implementation of AI, while 35% believe it is possible, indicating Radiology (n. 49: 84%), Pathological Anatomy (n. 33: 57%) and Dermatology (n. 12: 21%) as the three areas most at risk.

**Figure 7:**
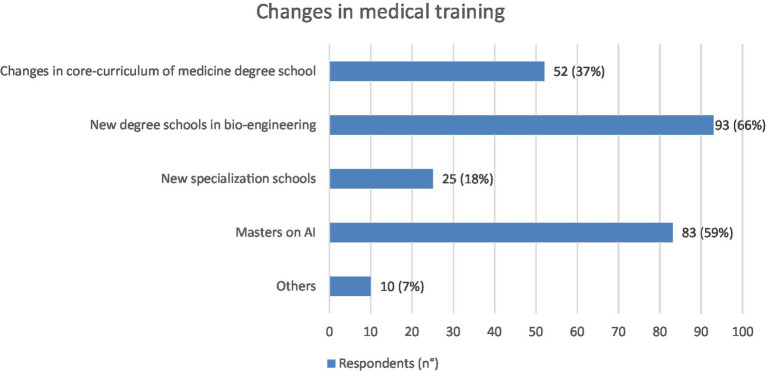
Graphic representation of the changes AI will cause in medical training according to the physicians interviewed (*N* = 140), each of whom was asked to indicate at least one cause. Values shown next to each bar represent the number of respondents who selected that option, with the corresponding percentage in parentheses. The most prevalent answers were “new Degree Schools” (*N* = 93) or “Master on AI” (*N* = 83).

The 87% of the interviewees believe that the medical training will have to be modified as a consequence of the impact of AI and, among these, the indications prevailing are the need to establish new degree schools in bio-engineering: 93 (66%), masters on the topic of AI: 83 (59%) and to modify the core curriculum of the Study Course in Medicine and Surgery: 52 (37%) (Figure 7).

**Figure 8:**
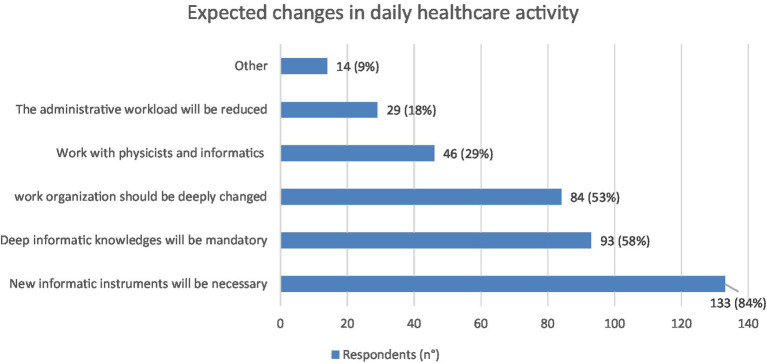
Graphic representation of the expected changes that the responders (*N* = 159) think will be caused in their daily healthcare activity by implementation of AI. Each respondent was asked to indicate up to three points. Values shown next to each bar represent the number of respondents who selected that option, with the corresponding percentage in parentheses. 133 respondents believe that they are necessary new informatics instruments and 93 that deep informatics knowledges will be mandatory.

As regards the potential changes induced by AI on medical care activities, the most numerous indications (n. 133: 84%) underline the need for new dedicated informatics tools, followed by that of having in-depth informatics knowledge requirements (n. 93: 58%) and finally by the belief that substantial changes in work organization will be necessary (n. 84: 53%) (Figure 8). With regard to the latter, a high number of indications (n. 120: 75%) focus on the need for spaces and informatic equipment dedicated to data processing, while 101 (64%) are those relating to the need to increase informatic staff and 96 (60%) are those that consider the increase in telemedicine activities to be likely.

**Figure 9:**
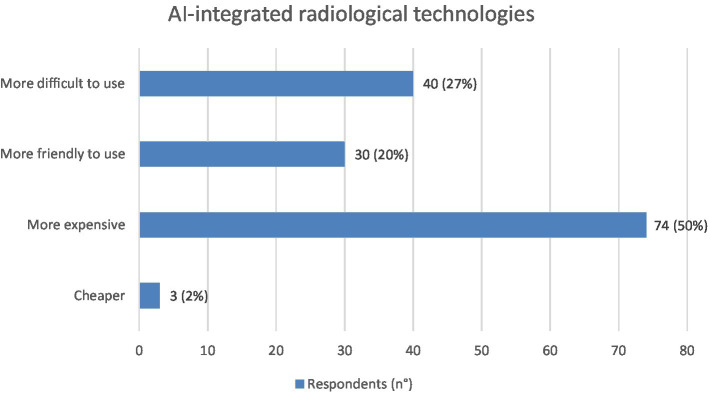
Graphic representation of how the respondents (*N* = 147) imagine new AI-integrated technologies. Values shown next to each bar represent the number of respondents who selected that option, with the corresponding percentage in parentheses. 74/147 interviewees believe that these new technologies will be more expensive.

The 91% of the interviewees believe that biomedical equipment manufacturers will have to modify their production as a consequence of the implementation of AI. Specifically, equipment integrated with an AI software will be more expensive for 74 (50%), more difficult to use for 40 (27%) and more friendly to use for 30 (20%) respondents (Figure 9).

The psychological feeling induced by the AI theme regarding one’s working future is represented by optimism in 54 (34%) of the interviewees, by worry in 48 (30%), by enthusiasm in 21 (13%), while indifference and anxiety are equally represented in about 15 (9%) (Figure 1).

#### 3.1.4 Section 4: expectations for the future role of AI in healthcare

156 (97%) respondents do not believe it is right that the treatment or diagnosis of a patient should be entirely entrusted to AI systems, while 141 (88%) believe it is right that AI can analytically support the Doctor in the diagnosis and treatment paths.

Finally, 156 (97%) of the interviewees believe that the intervention of a medical figure is always necessary.

### 3.2 Univariate analysis

Before proceeding with the multivariate regressions, some univariate analyses were preliminarily carried out between the independent variables and the regressors, in order to carry out an exploratory analysis of the regressors of interest.

Table 1 highlights that the majority of individuals, regardless of gender, believe that the training of the doctor will undergo changes due to the introduction of AI: 139 out of 160 individuals (about 87%). This value is fairly balanced between the two genders (69 men and 70 women). On the contrary, there is a notable difference in the group of those who think that the training path will not undergo changes, where the male group has more individuals (15/21 = 71.4%) than the female group (6).

**Table 1:** Relationship between possible changes in the training path of the doctor and the gender of the interviewee.

Training	Gender (M)	Gender (F)	Total
AI does not affect medical education	15	6	21
AI affects medical education	69	70	139
Total	84	76	160

Regardless of gender, most respondents believe that medical training will change with the AI introduction.

Table 2 shows that women tend to be more anxious or concerned about the impact of AI on their future careers than men: 48.05% of women (37/77) express anxiety or concern, versus 29.76% of men (25/84). Men, on the other hand, are more optimistic or enthusiastic about AI: 53.57% of men (45/84) feel positive about AI, versus 38.96% of women (30/77). The “indifferent” respondents appear to be almost evenly distributed between men and women.

**Table 2:** Relationship between sensations about the impact of AI on future doctor’s career and the interviewee’s gender.

Sensation	Gender (M)	Gender (F)	Total
The feeling about AI is Anxiety or Concern	25	37	62
The feeling about AI is Indifference or Other	14	10	24
The feeling about AI is Optimism or Enthusiasm	45	30	75
Total	84	77	161

The prevailing feelings in the female gender are anxiety and concern. On the other hand the prevailing feelings in the male gender are optimism and enthusiasm.

In summary, there seems to be a general trend where men are more likely to see AI as a source of optimism, while women tend to perceive it with greater anxiety or concern.

Table 3 shows that the majority of doctors, regardless of age group, do not believe that AI will reduce medical-legal liability (130 out of 160 Doctors, equal to 81.25%). However, there are 30 doctors (18.75%) who think that AI will have a positive impact in this sense. In particular, the youngest group (group 1, born between 1990 and 2000) is more likely to think that AI can ease medical-legal liability than older age groups: 11/69 (15.9%) in this group share this opinion. On the contrary, among doctors born before 1960 (group 5), only 1/9 (11.1%) believe that AI will reduce medical-legal liability.

**Table 3:** Relationship between the medico-legal liability following the IA introduction and the year of birth of the interviewee.

Medico-legal liability	1990–2000	1990–1980	1970–1980	1960–1970	before 1960	Total
Legal liability is not alleviated by the use of AI	58	30	20	14	8	130
Legal liability is alleviated by the use of AI	11	6	6	6	1	30
Total	69	36	26	20	9	160

The majority of the doctors, regardless of age group, do not believe that AI will reduce the medical-legal liability.

This difference may reflect a greater openness to technological innovation among younger doctors, who may be more familiar with the use of AI and therefore view it more positively than older doctors, who may be more skeptical or less exposed to this technology.

### 3.3 Multivariate analysis

Based on the results obtained with the univariate analysis, we decided to proceed with a multivariate analysis that considered the role of: age, gender, workplace and type of specialization on the dependent variables identified in section 2.1: changes in the doctor’s training path, feelings about AI and future career, professional liability and AI. Such a multivariate analysis is aimed to assess whether the univariate preliminary impressions were confirmed.^2^ To be noted that the main goal of the analysis is to explore potential patterns in the data. Therefore, results are reported without multiplicity corrections, consistent with an exploratory framework.

The results of the multivariate analysis are reported in Table 4. From Panel A it emerges that gender has a 5% statistically significant association with the belief that doctors’ training path will be modified due to AI, with women being, on average, 13.3 percentage points more likely than men to hold this belief, after controlling for all the other regressors. Instead, physicians practicing in a region different from Emilia Romagna are, on average, 12.9 percentage points less likely (at 10% significance level) to believe that medical training will undergo changes due to the introduction of AI. The year of birth and being a radiology specialist or resident are not statistically significant, therefore there does not seem to be a different perception about the effect of AI on training between Doctors and radiology trainees and other types of specialists or trainees or on the basis of age. Panel B and Panel C investigate physicians’ different feelings (negative, neutral, positive) about their own future career due to AI and show that women are more likely (at 5% significance level) to have negative feelings (i.e., anxiety or worry), whereas doctors outside Emilia Romagna are marginally more likely to have positive feelings (i.e., enthusiasm or optimism). Specifically, being female is associated with a 16.6 percentage point increase in the probability of expressing negative feelings and with a 17.2 percentage point decrease in the probability of expressing positive feelings. On the contrary, practicing outside Emilia Romagna is associated with a 14.5 percentage point decrease in the probability of expressing negative feelings and with a 15.5 percentage point increase in the probability of expressing positive feelings. Again, the year of birth and being a radiology specialist or resident do not show a statistically significant association. Hence, female physicians are more likely than men to believe in a change in their training path due to AI and to be more pessimistic about AI consequences in their future career, confirming impressions emerging from Tables 1, 2. Finally, Panel D shows that gender is the only variable showing statistical significance with professional liability, implying that women are, on average, 12.9 percentage points less likely than men to feel that AI will ease medical-legal liability. This evidence does not confirm impressions emerging from Table 3, which, instead, suggest a relation between professional liability and physician’s age. Overall, the analyzed independent variables partially explain the variations in physicians’ opinions. The low pseudo R-squared value in the feelings and professional liability models suggest that, beyond the analyzed variables, other factors may influence the different spectrum of sensations related to AI. The other factors, not analyzed in this study, are likely to be related to individual personality traits or differences connected to degree of integration of AI into the work environment and daily lives of respondents.^3^ However, for the ordered logit model, used to investigate physicians’ different feelings about their own future career due to AI, the Brant test does not reject the proportional odds assumption, confirming that the ordered logit specification is appropriate. Moreover, in the logit models for Educational path and Liability, the Hosmer–Lemeshow goodness-of-fit test indicates no evidence of lack of fit, suggesting that the logistic regression model provides an adequate representation of the data.

**Table 4:** Multivariate analysis.

Panel A—educational path									
Coef	Cat.	Beta	Marginal effects	Odds ratio	95% CI		GVIF	Df	GVIF^(1/(2 × df))
(Intercept)		1.810** (0.758)	Nc	6.108	1.381	27.005			
Year of birth	2	0.279 (0.606)	0.035 (0.074)	1.322	0.403	4.332	1.131	3	1.021
	3	0.648 (0.853)	0.073 (0.085)	1.912	0.359	10.186			
	4	16.749 (1177.301)	Nc	Nc	Nc	Nc			
Gen (F)	1	1.103** (0.540)	0.133** (0.061)	3.013	1.046	8.680	1.029	1	1.015
No ER	1	−1.019* (0.549)	−0.129* (0.070)	0.361	0.123	1.059	1.146	1	1.070
Radiologist	1	−0.284 (0.715)	−0.033 (0.079)	0.753	0.186	3.054	1.066	1	1.033
Pseudo R-squared	16.36%								
Observations	131								
Hosmer–Lemeshow Test	Chi2 = 1.80, *p*-value = 0.987								

Panel B—feelings									
Coef	Cat.	Beta	Odds Ratio	95% CI		Brant test *p*-value	GVIF	Df	GVIF^(1/(2 × df))
(Intercept)	1|2	−0.140 (0.453)	0.869	0.358	2.113				
	2|3	0.498 (0.454)	1.646	0.675	4.011				
Year of birth	2	0.237 (0.411)	1.268	0.567	2.835	0.812	1.255	3	1.039
	3	0.518 (0.472)	1.678	0.665	4.233	0.947			
	4	0.311 (0.463)	1.364	0.551	3.380	0.453			
Gen (F)	1	−0.721** (0.309)	0.486	0.265	0.892	0.311	1.026	1	1.013
No ER	1	0.652* (0.358)	1.919	0.951	3.871	0.724	1.272	1	1.128
Radiologist	1	0.320 (0.398)	1.378	0.631	3.005	0.248	1.063	1	1.031
Pseudo R-squared	3.05%								
Observations	161								
Brant test for all variables	Chi2 = 3.51, *p*-value = 0.742								

Panel C—feelings (marginal effects)				
Coef	Cat.	Feelings = Negative	Feelings = Indifference	Feelings = Positive
Year of birth	2	−0.054 (0.093)	−0.001 (0.004)	0.055 (0.095)
	3	−0.114 (0.100)	−0.007 (0.011)	0.121 (0.109)
	4	−0.070 (0.103)	−0.002 (0.006)	0.072 (0.108)
Gen (F)	1	0.166** (0.070)	0.007 (0.008)	−0.172** (0.072)
No ER	1	−0.145* (0.076)	−0.010 (0.010)	0.155* (0.083)
Radiologist	1	−0.074 (0.093)	−0.001 (0.003)	0.075 (0.092)
Pseudo R-squared	3.05%			
Observations	161			

Panel D—professional liability									
Coef	Cat.	Beta	Marginal effects	Odds ratio	95% CI		GVIF	Df	GVIF^(1/(2 × df))
(Intercept)		−1.006* (0.583)	Nc	0.366	0.117	1.145			
Year of birth	2	−0.042 (0.582)	−0.006 (0.076)	0.959	0.307	2.997	1.298	3	1.044
	3	0.440 (0.627)	0.067 (0.100)	1.553	0.454	5.308			
	4	0.466 (0.608)	0.071 (0.097)	1.593	0.484	5.245			
Gen (F)	1	−0.900** (0.441)	−0.129** (0.060)	0.406	0.171	0.964	1.014	1	1.007
No ER	1	0.094 (0.501)	0.014 (0.074)	1.098	0.411	2.934	1.322	1	1.150
Radiologist	1	−0.370 (0.497)	−0.058 (0.082)	0.691	0.261	1.830	1.074	1	1.037
Pseudo R-squared	4.31%								
Observations	160								
Hosmer–Lemeshow Test	Chi2 = 7.70, p-value = 0.464								

The table shows the multivariate regression analysis for the three identified dependent variables: educational path, feelings, and professional liability, compared to the selected regressors: year (classes merged to balance the sample size), gender (dummy for being female), region of work (dummy for practicing outside Emilia Romagna), and specialty (dummy for being a specialist or resident in radiology vs. in other specialist areas). In Panel A the dependent variable is represented by the “training”, which indicates whether the Doctors believe (1) or do not believe (0) that their training path will be modified due to AI. In Panels B and C the dependent variable is “Feelings” of the doctors about their future work, with the following codings: (1) Negative feelings (anxiety and concern), (2) Indifference or other neutral feelings, (3) Positive feelings (enthusiasm or optimism). In Panel D the dependent variable is “Professional liability “, which indicates whether the doctors believe (1) or do not believe (0) that AI will ease medical-legal liability. A logistic model was used for educational path and professional liability, while an ordinal logistic model was used for feelings. For each model, we reported betas (with standard errors in parentheses), marginal effects (with standard errors in parentheses), the odds ratio with their 95% CI, GVIF, pseudo R-squared and the number of observations. For the ordered logit model also the Brant test for proportional odds check is reported, while for the logit models also the Hosmer–Lemeshow goodness-of-fit test is reported (estimated with 10 groups as commonly recommended in the literature). ***, **, and * represent significance at 1, 5, 10% levels, respectively.

Table 5 reports the results of a sensitivity analysis conducted along two main lines. First, we exclude the No ER variable from the specification of the models. Second, we adopt two alternative redefinitions of the Feelings variable as a binary indicator: (i) the new variable “Negative feelings” is equal to 1 when Feelings about AI and Future Career are negative, and equal to 0 when they are positive or show indifference; (ii) the new variable “Positive feelings” is equal to 1 when Feelings about AI and Future Career are positive and equal to 0 when they are negative or show indifference.

**Table 5:** Sensitivity analysis.

Sensitivity analysis								
Model		Logit	Ordered logit			Logit	Logit	Logit
Variables	Cat.	Educational path	Feelings = Negative	Feelings = Indifference	Feelings = Positive	Professional liability	Negative feelings	Positive feelings
Year of birth	2	0.064 (0.074)	−0.016 (0.091)	−0.001 (0.004)	0.016 (0.095)	−0.009 (0.075)	−0.043 (0.101)	0.064 (0.103)
	3	0.120* (0.072)	−0.053 (0.098)	−0.003 (0.008)	0.057 (0.106)	0.061 (0.093)	−0.121 (0.108)	0.129 (0.118)
	4	Nc	0.000 (0.099)	0.000 (0.003)	−0.000 (0.103)	0.064 (0.088)	−0.036 (0.111)	0.099 (0.115)
Gen (F)	1	0.137** (0.062)	0.169** (0.070)	0.007 (0.008)	−0.176** (0.073)	−0.130** (0.060)	0.192** (0.075)	−0.145* (0.078)
No ER	1						−0.158* (0.084)	0.148* (0.090)
Radiologist	1	−0.0576 (0.072)	−0.101 (0.093)	−0.001 (0.005)	0.102 (0.090)	−0.055 (0.080)	−0.101 (0.098)	0.032 (0.099)
Pseudo R-squared		0.066	0.020	0.020	0.020	0.043	0.053	0.030
Observations		131	161	161	161	160	161	161

The table shows marginal effects of the sensitivity analysis for the multivariate analysis.

Columns 3–7 drop the No ER variable, while columns 8–9 reclassify the feelings trichotomy in a binary variable: in column 8 Negative feelings is equal to 1 when Feelings about AI and Future Career are negative, and equal to 0 when they are positive or show indifference; in column 9 Positive feelings is equal to 1 when Feelings about AI and Future Career are positive, and equal to 0 when they are negative or show indifference. Standard errors are reported in parentheses, ***, ** and * represent significance at 1, 5, 10% levels, respectively.

The results of the sensitivity analysis confirm the significant role of Gender across all models. When the No ER variable is excluded, the Year of birth variable becomes marginally significant, for the cohort born between 1970 and 1980, in the model explaining the Educational path. This change is likely due to the exclusion of No ER, which may have absorbed part of the variation previously attributed to other covariates.

## 4 Discussion

AI is spreading more and more, both in many human activities and in healthcare system, and the field of diagnostic imaging is particularly involved by its growth (Kelly et al., 2022; Van Leeuwen et al., 2021; Van Leeuwen et al., 2024). Despite the clear evidence of benefits related to its implementation, many physicians worry about ethical, legal, employment and professional changes that AI is going to induce (Aung et al., 2021; Castagno and Khalifa, 2020; Petersson et al., 2022; Waymel et al., 2019; Apell and Eriksson, 2023; European Society of Radiology, 2019).

The aim of this work has been to directly test with a voluntary survey the work impact of AI on a group of Italian physicians belonging to specialties in which the impact of AI is considered more relevant such as Radiology, Cardiology and Pathological Anatomy. The aim of this survey was to understand whether and why radiologists and other physicians are concerned about AI.

The response rate to the survey was of 48%, which, on the basis of what is reported in the literature, can be considered quite satisfactory. Indeed, the use of questionnaires or interviews aimed to analyze the perception of healthcare personnel on the topic of AI has already been the subject of reports in the literature but with more limited samples. Our response rate is similar to that detected by Waymel et al. (2019) (43.8%) in electronic survey sent to French radiologists. On the contrary only 2.8% of European Society of Radiology (ESR) Members completed the EuroAIM survey about the impact of AI on radiology, the results of which are published in a 2019 statement (European Society of Radiology, 2019). The study by Petersson et al. (2022), aimed only at “healthcare leaders” includes 26 interviews, while that of Castagno and Khalifa (2020)consists of the responses to a questionnaire of 8 questions provided by a sample of 98 healthcare workers, with a response rate of 1.3%, therefore significantly lower than the data found in this work. Another study, conducted by Apell and Eriksson (2023), was developed with the interview-questionnaire method with 21 expert operators in the field of AI in healthcare and 25 public bodies and companies involved in various capacities and degrees in the development of AI systems in healthcare.

Data relating to competence and personal familiarity with the topic of AI show that 47% of the respondents declared a fair level of knowledge, 30% a poor level, and 15% a good level. This data suggests that AI-related skills are gradually spreading, albeit slowly. The presence in the sample of a large proportion of young or relatively young doctors, for whom digital and informatics skills are generally more widespread than among older medical professionals, likely contributes to the overall 65% who consider their skills to be excellent, good or fair.

A similar 2020 report by Castagno and Khalifa (2020), which included also non-medical healthcare professionals, found that 64% of respondents reported no competence in AI, with around 88% stating that they were unaware of the difference between deep learning and machine learning. The issue of still limited AI competence among healthcare professionals is also highlighted by other sources. In particular, Apell and Eriksson (2023) partially attributes to this phenomenon the difficulty some AI system manufacturers face when trying to engage with knowledgeable healthcare personnel, understand their needs, and receive authoritative guidance on research and development directions to pursue.

From the answers analyzed it emerges that only the 19 and 7% stated to carry out often or daily, respectively, clinical activities involving the use of AI systems and only the 12 and 1% declare to have conducted research activities related to AI applications often or on a daily basis, respectively. At this point, the question is: *do we not use, or have the awareness to use, in our work but also and especially in our daily lives tools whose operation make use of AI?*

These results are actually quite surprising since many healthcare devices, especially in the radiological field (e.g., MRI) but also non-healthcare devices (think of systems like SIRI or Alexa), already use AI systems for the production of images or speech, but it is likely that some of the interviewees may not have been aware of this. Even in Castagno and Khalifa (2020), 63% of the interviewees declared that they had never used AI systems, not only at work but also in everyday life. The data reported show that there is a lack of awareness of the presence of AI in everyday work or life.

The data relating to research activity on the topic of AI are instead much more in line with expectations, since it is likely that such activities are currently limited to a small percentage of the sample, presumably to personnel dedicated to carrying out research for institutional or curricular tasks.

Radiology, Pathology, and Neurology were indicated from responders as the three Medical Specialties most predictably affected by the impact of AI.

This perception is certainly supported by extensive evidence in the literature, if we consider that a systematic review carried out in 2022 had already identified 535 papers on the topic of AI applied to Radiology (Kelly et al., 2022) and that already in 2021 there were more than two hundred CE-marked AI products for radiology on the market for clinical use (Van Leeuwen et al., 2021; Van Leeuwen et al., 2024). This is also understandable since modern radiology is based almost exclusively on digital images (think of CT and MRI) which lend themselves well to the processing and calculations needed by AI software. A little more surprising is the indication relating to Pathology, whereas the prevalence of the literature identifies Dermatology and Cardiology as the disciplines most involved in AI (Vidali, 2024), although some contributions describe important experiences and promising results in the application of AI in the pathological field (Steiner et al., 2018).

However, although awareness of the extent to which AI is already integrated into daily clinical activity and life in general may be limited, 98% of respondents nevertheless recognize the potential benefits of AI in the healthcare sector, in accordance with the literature (Van Leeuwen et al., 2021; Van Leeuwen et al., 2024; Stogiannos et al., 2025). The attitude of healthcare professionals towards AI is probably changing over the years, if we consider that while our questionnaire showed that 98% of operators believed that it would bring benefits to the healthcare world just a few years ago, Castagno and Khalifa (2020) showed that only 50% of operators considered it useful for their work, while 40% considered it more dangerous than a nuclear device.

The reduction of medical errors is certainly the most awaited and the most concrete of the benefits expected from AI, as already reported in the literature where the benefit, in terms of diagnostic accuracy, particularly for diagnostic purposes, clearly emerges. These results agree with that obtained by Waymel et al. (2019) in their survey submitted to French Radiologists, and in the survey of Castagno and Khalifa (2020), submitted to the Healthcare Staff of the Royal Free Hospital in London.

The problem of medico-legal consequences, the first of the expected negative effects, is certainly not marginal since, faced with systems that can make decisions in an almost autonomous way, it is necessary to establish with certainty whether the consequences of the decisions taken using AI fall under the responsibility of the AI productor or the doctor who uses it or both (Vidali, 2024, Castagno and Khalifa2020, Petersson et al., 2022). The recent WHO document setting forth the ethical principles for consenting to the use of AI for health also reiterates the need to define accountability and transparency and to ensure that AI is used by appropriately trained individuals. Adequate compensation mechanisms must be established for those who suffer harm caused by AI (WHO, 2024).

The worsening of the doctor-patient relationship is an understandable element, since the interposition of an AI system between doctor and patient increases the communication distance making it more difficult since, in addition to the already complex aspects of a strictly technical-scientific nature, it is necessary for the patient to also be informed of the presence of an external “non-human” system whose intervention could be decisive in the choices of the diagnosis and treatment path. Even authors of the philosophical and psychological area (LaRosa and Danks, 2018) underline the important consequences of the interposition of AI systems in the doctor-patient relationship, with consequences not only of medical-legal liability but also of a relational and social nature. Technological development and the spread of AI-driven devices in healthcare have been associated with the dehumanization of the doctor-patient relationship, lacking empathy—the inherent ability of the human species to share and understand the patient’s illness and suffering. This problem has emerged particularly as a consequence of the introduction of AI-driven robots in healthcare. On the other hand, however, as reported in a recent bioethics study (Sirgiovanni, 2025), in some cases empathy itself in the doctor-patient relationship can lead to distorted reasoning and decisions, as demonstrated by psychological and philosophical research. Therefore, engineers considering creating affective AI systems should therefore consider the risks of incorporating empathy into such systems, but instead foster feelings such as sympathy and compassion, which ethicists consider more useful in clinical contexts. A recent study in the literature highlights the concern of patients that the increasingly important role of AI in the medical field could influence doctor-patient interaction, underlining the need for guidelines on the integration of AI into clinical processes to ensure patient-centered care (Witkowski et al., 2024).

In the present study, only 25% of the interviewees consider relevant the problem of the increase in healthcare costs potentially induced by AI, which instead is well highlighted by observers of the non-medical area, such as “healthcare leaders” or “corporate decision makers” as reported by Petersson et al. (2022) and Apell and Eriksson (2023). The increase in healthcare costs is mainly due to the technology needed to support the introduction of AI in the healthcare world which at the time of the introduction of AI is certainly a cause of huge expenses, however other authors (e.g., Holdsworth and Zaghloul, 2022) report expectations of large savings in the healthcare world (212.4 billion euros/year in Europe) once AI is implemented in the healthcare area.

The move toward increasingly personalized medicine requires high-level technology and professionals capable of managing and producing significant amounts of real-time data from lifestyle, environmental, and genetic factors. In the work of Tan et al. (2025a) AI is proposed as a tool to produce and integrate a huge amount of patient data from different sources representing an example of how technology can be useful in managing all patient information and obtaining a comprehensive, patient-centered view. This requires to use advanced technology and professional support within multidisciplinary teams, and at the same time, can lead to disparities in treatment methods, excluding poorer populations from the benefits of personalized medicine. Ethics and privacy are the issues highlighted in the work by Tan et al. (2025a) that must be addressed if we are to reap the enormous benefits of actively integrating AI into the patient care journey.

A similar study by Tan et al. (2025b), published in Frontiers in Digital Health, addresses the issue of the lack of access to advanced diagnostic tools and healthcare professionals in the Global South, as well as the problem of outdated infrastructure, resulting in a lack of homogeneity in the use of AI innovations in patient care. This is also an element reiterated in the WHO document which, among its ethical principles, includes ensuring inclusiveness and equity, that is, appropriate and fair use, regardless of the income of individual countries (WHO, 2024).

Another very important point that can influence the mood of healthcare professionals towards AI is represented by the impact of AI on medical employment.

Indeed, as already stated by Castagno and Khalifa (2020), many physicians clearly worry about possible negative consequences of AI, first of all in the field of medical employment. Most of them, in fact, believe that the role of physician could be dramatically reduced by the diffusion of AI, that could lead to the appearance of new professional healthcare figures, such as physicists or engineers dedicated to AI system implementation, as already reported by Petersson et al. (2022), in their qualitative interview study with healthcare leaders in Sweden. About the half of our responders, in fact, believe that these new healthcare figures might cause the reduction of medical employment chances that, together with the diagnostic capabilities of the AI software, could lead to the reduction of some medical specialties, one of which is just radiology.

Tan et al. provide an example of new healthcare professionals in the era of AI-based precision medicine in a recent work, by introducing data scientists working on tumor boards, experts in machine learning, data analytics, and bioinformatics. These professionals have the ability to synthesize and analyze diverse data sets generated during a patient’s diagnostic journey, particularly in oncology, providing practical information on outcomes and treatment decisions that can optimize treatment decisions (Tan et al., 2025b).

The fear of the impact on employment is even more felt with the introduction of agentic artificial intelligence capable of offering a promising solution by autonomously managing complex healthcare task, reducing human error and enhancing efficiency using machine learning algorithms (Fuentes et al., 2025). However, knowing the mechanisms well helps to recognize how agentic AI allows us to revolutionize healthcare by enhancing administrative efficiency, improving decision making, streamlining operations and supporting economic sustainability. On the contrary, by automating many administrative processes it can reduce the burden of physicians and make work more efficient, patient-centered and more sustainable.

The possible reduction in the medical employment rate, as a consequence of AI, is a real problem not only in the healthcare world, if we consider that the 2020 World Economic Forum study “The future of jobs Report” (Future of Jobs Report, 2023) predicted that AI could replace up to 85,000,000 jobs, although it should be emphasized that, in the face of the replacement of some healthcare activities by AI, the implementation of the latter will necessarily lead to the creation of new job categories (DL and ML specialists, AI solution architects, cybersecurity workers, etc.) which, however, will likely not necessarily belong to healthcare roles (Vidali, 2024).

The transformation of healthcare roles and the emergence of new managerial, physical and IT professionals as a result of AI implementation is certainly a current issue, which is well known to a high percentage of interviewees (58%), as well as to healthcare leaders (Petersson et al., 2022). These leaders also foresee changes to healthcare hierarchies, established roles and the division of labor, which could lead to organizational resistance to innovation. The expected change in the professional figures sought, with a greater emphasis on engineering and IT skills, also provides strong evidence (87% of respondents) that the training of young doctors needs to be modified to include the required engineering and informatics skills.

Indeed, despite the increasingly crucial role of AI in healthcare, both in patient management and in treatment planning, this awareness clashes with the perception of a lack of knowledge and technical skills in using AI. This is evident across various professional levels and levels of education. We can say that a discrepancy emerges between awareness of its increasingly important role and the technical preparation required to properly manage this tool (Vanamali et al., 2025).

The topic of training and updating of operators is indeed very relevant, to the point that even the Italian Ministry of Health underlines the need to prepare not only university or post-university training modules to improve the knowledge and skills of healthcare workers on the topic of AI, but also methodological elements on AI already in secondary school programs.

Other authors (Petersson et al., 2022; Goyal et al., 2024; Doherty et al., 2024) also underline the need of deep changes in training paths, not only for medical personnel but also for technical-nursing and administrative personnel, claiming the inclusion of AI also in secondary schools with the aim of creating useful knowledge bases not only for those who will undertake healthcare paths but to increase and spread knowledge on the topic of AI also by the general population who could be its future users.

Several studies in the literature highlight the importance of training and the positive effect that increased knowledge has on acceptance and awareness of the risks and benefits associated with introducing AI into healthcare processes. For example, Catanese et al. (2024) underline as acquiring a profound understanding of AI fundamentals can aid radiologists in evaluating the risks and benefits associated with AI. The medical students who participated in the study of Farooq and Usmani (2025) believed that the AI integration into medical education improves medical concepts and learning experience and will have a positive impact on patient outcomes, healthcare delivery and future practices. Also in the work of Li et al. (2025) the survey reveals that the radiologists, residents, and medical students demonstrated a positive attitude towards the implementation of AI in radiology education and a framework of AI-assisted radiology education training platform was recommended. The importance of AI training programs is highlighted in several studies that address this topic from different professional perspectives. A recent review (Shishehgar et al., 2025) suggests that training should empower healthcare students, particularly nursing, with confidence about AI-based technology concepts, implications, challenges, and ethical considerations. Syeda et al. (2025) highlight the need for structured AI education in medical curricula to bridge knowledge gaps and prepare future healthcare professionals for AI-driven practice. Drevitska et al. (2024) argue that AI tools like ChatGPT are valuable resources in medical education and practice but their adoption is accompanied by caution due to inherent limitations and the necessity of critical, careful use. In addition, the study underscores the need for further research and the integration of AI ethics into medical curricula.

Also Huisman et al. (2021) concluded that limited AI-specific knowledge levels among radiology residents and radiologists are associated with fear, while intermediate to advanced AI-specific knowledge levels are associated with a positive attitude towards AI. Therefore additional training may improve clinical AI adoption. The integration of training courses with notions to support knowledge and understanding of AI will therefore allow for greater awareness in all job categories, both in the healthcare world and beyond and the resulting increased awareness will likely help reduce fears about AI.

The topic of AI literacy is widespread and requires modules of varying complexity aimed at different levels of educational attainment, from basic education to medical training in degree courses. Overall, the literature is consistent and emphasizes the important role of AI training in increasing awareness of the use of these new tools, while also addressing ethics and medical-legal issues that enable more responsible and informed physicians in practice. The pathways and tools for integrating student training remain unclear, requiring national and international comparisons and adjustments.

Few studies consider the patient’s perspective, as reported by Erul et al. (2025) in a study analyzing surveys administered to cancer patients. Patient perceptions and attitudes toward AI depend on their level of education. It is important to establish targeted educational interventions to improve understanding of the role of AI and patient acceptance. Establishing shared national guidelines is useful for improving patient acceptance (Erul et al., 2025).

Our data about the psychological feeling induced by AI do not differ significantly from Castagno and Khalifa (2020) results, which reported approximately 30% of patients as extremely to slightly worried and 70% as not at all worried.

Indeed, even though a large part of the interviewed physicians is positive or enthusiastic in regard to AI, about half of them, in particular females, are clearly worrying or anxious about it. Are they right? We do not know, since this study is not tailored to answer this question and we believe that only further studies in the next years might answer it. The fact is that in more recent literature reports it is described that frequent AI use was associated with an increased risk of radiologist burnout, particularly among those with high workload or lower AI acceptance (Liu et al., 2024).

Finally, 98% of the interviewees believe that the intervention of a medical figure is always necessary, the 97% of the sample does not believe it is right that the treatment or diagnosis of a patient should be entirely entrusted to AI systems, while 88% believe it is right that AI can analytically support the Doctor in the diagnosis and treatment paths. On this topic, an interesting element of reflection is provided by the work of Petersson et al. (2022) in which the “healthcare leaders” fear the possibility of an impoverishment of traditional medical culture in the new medical generations, as a consequence of the possibility of delegating or abdicating the traditional processes of diagnosis and therapeutic decision to the choices made by AI systems.

However, at present, we agree with Langlotz (2019) stating that “Will AI replace Radiologists?” is the wrong question. The right answer is: Radiologists who use AI will replace Radiologists who do not.” We also agree with the assumption that “the final decision should rest with a human,” (Kahraman et al., 2024) as AI should collaboratively assist healthcare professionals by reducing the workload during clinical activities and working transparently and systematically.

The results of the multivariate analysis about the feelings of doctors about their future work show that the variable gender is statistically significant. In particular, women tend to have more negative feelings about their future career than men and non-Emilia-Romagna doctors tend to have slightly more positive feelings than Emilia-Romagna doctors (although this latter effect is marginal).

The results of the multivariate analysis in which the dependent variable is whether or not doctors believe that their training path will be changed due to AI indicate that women are more likely than men to believe that the training path will undergo changes due to AI and that Doctors outside of Emilia-Romagna tend to believe (although marginally) that their training path will undergo fewer changes than those operating in Emilia-Romagna.

The results of the multivariate analysis in which the dependent variable is whether or not doctors believe that AI will alleviate medical-legal liability have highlighted that women are less likely than men to believe that AI will ease medical-legal liability.

Overall, gender appears statistically significant on all three variables considered.

The moderate role of gender differences in relation to technological advancement and the use of AI is the subject of numerous studies in the predominantly psychological literature. One example is the work by Russo et al. (2025), in which the authors explore gender-related differences in access to and use of AI, and in particular AI-related anxiety. The study shows, similarly to our own findings, that women have higher levels of anxiety regarding AI adoption, lower attitudes toward AI, lower use, and less perceived knowledge. A significant negative relationship was found between AI anxiety and positive attitudes toward AI. These differences, however, are evident at low levels of anxiety, while at high levels of anxiety, gender differences tend to level out, so much so that the authors refer to “gender differences leveler.” The same authors find a possible explanation for the differences, particularly in AI anxiety, in light of the digital gender gap and gender-based socialization processes that shape women’s interaction with technologies or by gender stereotypes that discourage women from delving into STEM fields and acquiring technological skills. Strategies are also proposed to bridge this gap in the digital world, such as ensuring equal access to AI training and education, as well as supporting women in leadership positions in AI sectors. Here, the need for a literacy process based on equitable educational and practical modules emerges once again, seeking to stimulate and strengthen AI aptitude.

A study of high school students in Ghana investigates gender differences in AI-based tools that have become integral to teaching and learning in HE schools. Gender has been found to be a determining factor in the use of AI-based tools and responsible for different perceptions regarding technological innovation. The author urges the dissemination of school policies aimed at greater female involvement in the use of AI-based tools (Ampong, 2023).

Other authors such as Grassini and Ree (2023) have also focused on understanding the factors that influence attitudes towards AI, emphasizing the importance of cultural context and gender, providing a basis for developing tools to implement the diffusion of AI.

Even in studies not strictly related to healthcare, gender differences related to AI perceptions emerge, for example in the field of marketing strategies. A Korean study by Ahn et al. (2022) analyzed gender stereotypes in the evaluation of AI recommendations. Male AI agents were recognized as having greater competence, while female agents were perceived as having greater warmth. Furthermore, more proactive attitudes were obtained when male AI agents proposed utilitarian products and female agents proposed hedonic products.

Another study by Mashburn et al. (2025) highlights gender differences in the use of generative AI in clinical practice and healthcare decision-making. Women showed greater improvement than men after using ChatGPT in decision-making in occupational medicine cases. The authors emphasize the importance of gender-sensitive training to improve clinical performance.

The equitable use and access to AI is reiterated by the WHO which underlines: “AI is designed and shared to encourage the widest possible, appropriate, equitable use and access, irrespective of age, sex, gender identity, income, race, ethnicity, sexual orientation, ability or other characteristics” (WHO, 2024).

The results obtained regarding gender differences confirm the literature on the role of the TAM and UTAUT models in technology acceptance and, in particular, confirm the role of gender as a moderator in the UTAUT models. Not only can gender differences play a role in technology acceptance and use, but they can also influence the role of the main constructs underlying the TAM and also presents into the UTAUT models. For example, perceived usefulness or perceived ease of use may differ depending on gender, and this can influence acceptance (Afonso et al., 2012; Lee et al., 2025).

However, analyzing the performance of the multivariate analysis models we constructed, particularly the pseudo R-squared values and the likelihood ratio test (LRT), it has emerged that the analyzed variables only partially explain the variations in physicians’ opinions. Indeed, only the educational path model appears to have a good ability to explain the variations in physicians’ responses, recognizing gender as a significant variable and work residence as a nearly significant variable. The other two models show a limited explanation and are not statistically significant.

The low pseudo R-squared value in the feelings and professional liability model, suggests that, beyond the analyzed variables, other factors may influence the spectrum of sensations related to AI. There are likely other factors we have not considered that may influence AI-related feelings and opinions about AI’s impact on medical-legal disputes. What we can think of is that respondents’ personality traits, but also the different ways in which AI is integrated into both the work environment and the respondents’ everyday lives, may play a role. Workloads should not be underestimated, nor should the respondents’ purely clinical or academic roles. These considerations pave the way for future studies that can verify our results and examine variables not considered in these analyses: it is desirable to introduce multivariate analyses that consider factors not analyzed in this work such as work context, personality, and workload.

## 5 Limitations of the study

This study presents some methodological limitations, among which the small size of the sample explored, which still makes the data partially significant. However, compared to other studies, it has the advantage of a high percentage of respondents to the survey. As no information was available for non-respondents, the risk of non-response bias cannot be formally assessed. Consequently, it cannot be excluded that respondents differed from non-respondents in demographic or professional characteristics, which may limit the generalizability of the findings.

An analysis of respondent characteristics reveals a bias in medical specialties, favoring in particular radiology (113 respondents) cardiology, and pathology. This may indeed constitute a bias, as opinions regarding other medical specialties are not captured. However, as confirmed by the literature, we can say that the responses most represented belong to the specialties in which the introduction of AI appears to have the greatest clinical and diagnostic impact.

Furthermore, most of the respondents come from Northern Italy (with 107 respondents based in Emilia-Romagna), and consequently, the survey results should be understood as a snapshot of the healthcare system in Northern Italy and not the entire country, a fact which, however, is supported by several studies in the literature analyzed. This may also be a limitation in multivariate analyses since the “no ER” sample still consists of people from regions of Northern Italy.

As a result, the findings primarily capture the perceptions and experiences of physicians working in this regional and professional context. While the main relationships observed in the analysis may offer valuable insights into broader patterns, they should not be generalized to all Italian physicians or medical specialties without caution. Future studies based on larger and more diversified samples would be useful to assess the external validity and the extent to which these findings can be replicated across professional groups and geographical areas.

Another potential limitation of our study is that all constructs measuring feelings and attitudes were assessed using single-items. Therefore, we were not able to compute internal consistency metrics (e.g., Cronbach’s *α*). Although some items are conceptually related, they were measured on different scales and cannot be combined into multi-item constructs.

The lack of response to some of the questions should be considered a limitation as the response rate varies between the different questions considered.

## 6 Conclusion

The study, although its observational nature does not allow for generalized conclusions, allows to define some thoughts.

First, the topic of AI is slowly entering the awareness, skills and care practices of medical personnel, particularly in the younger age groups.

The majority of personnel respondents expect positive effects from the implementation of AI, first of all the reduction of medical errors, but fear some negative effects, first of all the still uncertain consequences in terms of medical-legal liability.

Second, many operators interviewees fear that AI could take away space from medical employment, particularly in specialist areas such as radiology or that it could in any case lead to profound changes in the roles and organizational structures of the care process that generate feelings of anxiety or concern for the future in a large component of the sample. Optimism and concern about AI are almost equally represented.

Finally, the majority of the sample foresees the need for changes in the training and curricular path of the Doctor and the modification of spaces and technological tools aimed at making room for AI activities for which, although the figure of the Doctor remains indispensable, space will necessarily have to be given to new, non-medical figures dedicated to the AI theme.

In light of what is reported in the literature, our data confirm that awareness of the increasingly important role of AI must be accompanied by structural changes in training programs not only for doctors but also for other healthcare professions. This should include AI modules combined with internships to test the role of AI in practice. This would help fill technical gaps among healthcare professionals and reduce concerns, particularly regarding ethical considerations. In addition, the publication of official documents, such as the one recently published by WHO (2024), clearly defining the ethical and legal aspects would foster greater awareness of the new role of healthcare professionals using AI.

From the results of the estimates made through regression models, three main and rather robust results emerge. Gender is always statistically associated with the response given on the effects of AI regarding the impacts on training, feelings and litigation: women tend to be overall more pessimistic, predicting greater impacts on training, with a substantially negative feeling and with a lower probability of easing litigation. Secondly, it does not seem that the responses are correlated with the doctor’s specialty of the respondent.

Finally, the region in which the Doctor works, is also statistically associated with responses on training and feelings, although marginally, while it is not associated with the response on the effect of AI on litigation.

Overall, despite the limitations of this survey’s results, we can conclude that it has highlighted a greater awareness of the emerging impact of AI in our healthcare sample The introduction of new roles will effectively contribute to multidisciplinary patient management by enriching it with numerous data that will enhance care processes and relieving physicians’ burdens by supporting them in data collection and analysis. Proper training and information on the role of AI in care pathways is essential for the new management of the doctor-patient relationship in the era of precision medicine.

## Data Availability

The raw data supporting the conclusions of this article will be made available by the authors, without undue reservation.

## Footnotes

^1^We did not use the type of degree given that 98% had a degree in medicine and surgery, and the difference between specialist and resident given that it was captured by the year of birth.

^2^Multivariate analysis is conducted in Stata, which by default applies listwise deletion for missing data. Consequently, observations with missing information on any of the variables included in the model were excluded from the estimation. Given that most variables are binary or categorical, and that the dataset is cross-sectional (i.e., no temporal dimension), imputation procedures such as mean substitution or time-based interpolation would not be appropriate in this context.

^3^Please note that, in order for the questionnaire to be remain anonymous and to adhere to privacy regulation, we could not ask a set of more specific questions.
