# Supplementary material for: Physician perceptions of artificial intelligence in Northern Italy healthcare: a survey of fears and expectations
Source: Front Artif Intell. 2025 Nov 12;8:1624789. doi: 10.3389/frai.2025.1624789 (PMC12647062; doi:10.3389/frai.2025.1624789)
Supplement: Supplementary file 1 [file Data_Sheet_1.pdf]

Supplementary Material

Fig.1 Questionnaire: The questionnaire is divided into 4 sections. **Section 1**: personal or curricular data. **Section 2**: competence of the operators with the AI and the experiences already acquired on its use in the healthcare or research field. **Section 3**: benefits and the negative consequences of the implementation of AI, the opinions relating to the possible occupational implications of AI and the opinions relating to the changes, that the diffusion of AI could make necessary, both in the training and in healthcare activity; the last question is about the psychological status induced from the think of IA in the future working. **Section 4**: opinions about the legitimacy, ethics and medico-legal aspects related to the exclusive or complementary use of AI in diagnostic-therapeutic pathways.

**Section 1: PERSONAL DATA**
Year of birth:<br><br>• 1990 – 2000<br>• 1980-1990<br>• 1970-1980<br>• 1960-1970<br>• Before 1960
Gender:<br><br>• male<br>• female
Degree:<br><br>• Degree in Medicine<br>• Degree in Health Professions<br>• Other Degree<br>• Other
I am a specialist in:<br><br>• Radiology<br>• Cardiology<br>• Pathological Anatomy<br>• Other specialization<br>• None
I am Resident in:

- Radiology
- Cardiology
- Pathological Anatomy
- Other specialization
- None

Region of work location

- 21 Italian regions

Section 2: AI SKILLS

How do you rate your level of knowledge about AI?

- Excellent
- Good
- Fair
- Poor
- Very Poor

Do you perform or have you ever performed care activities that involve the use of AI?

- Never
- Rarely
- Often
- Daily
- Don't know

Do you or have you done research on AI applications?

- Never
- Rarely
- Often
- Daily
- Don't know

Which three medical specialties do you think would be most interested in using AI?

- General Surgery
- Gynecology
- Ophthalmology
- Dermatology
- Radiology

- Pathological Anatomy
- Cardiology
- Anesthesia
- Orthopedics
- Neurology

Section 3: AI IMPACT ASSESSMENT

Do you think AI will benefit the healthcare world?

- Yes
- No

If you answered yes to the previous question, what do you think are the ***3 main benefits*** *of using AI in medicine?*

- Improved doctor-patient relationship
- Reduction of healthcare costs
- Improved quality of care
- Reduction of medical errors
- Speeding up medical trials
- Acceleration of the discovery of new drugs
- Implementation of new biomedical techniques
- Increased professional earnings
- Other

What do you think could be the ***3 main negative consequences*** *of the use of AI in medicine?*

- Worsening of the doctor-patient relationship
- Increase in healthcare costs
- Worsening of the quality of care
- Increase in the complexity of the medical profession
- Disruption of the medical role
- Medical-legal consequences
- Reduction of earnings
- Other

From an employment perspective, what do you think could be the 3 main consequences of the use of AI in medicine?

- It will increase the possibility of medical employment
- It will reduce the possibility of medical employment
- It will lead to the replacement of the doctor by other professional figures
- It will lead to the extinction of some specialist areas
- Other

Do you think there are specialist areas at risk of extinction as a result of the use of AI?

- Yes
- No

If you answered yes to the previous question: what do you think could be the 3 specialist areas at greatest risk of extinction?

- General Surgery
- Gynecology
- Ophthalmology
- Dermatology
- Radiology
- Pathological Anatomy
- Cardiology
- Anesthesia
- Orthopedics
- Neurology
- Other

Do you think that the spread of AI will lead to changes in the training of doctors?

- Yes
- No

If you answered yes to the previous question: how do you think AI will change the training path of doctors?

- It will lead to changes in the core curriculum of the Medical Degree Course
- It will lead to the establishment of new degree courses with a focus on bio-engineering and computer science
- It will lead to the creation of new specialization schools
- It will lead to the creation of Masters on AI
- Other

How do you think the implementation of AI will change the healthcare activity of doctors? (indicate up to 3 points)

- It will require in-depth computer science knowledge to carry out the profession
- It will involve the availability of new dedicated computer science tools
- It will involve the need to always work with physicists and computer science experts
- It will involve substantial changes in the organization of work
- It will reduce the bureaucratic burden
- Other

What do you think could be the 3 main changes in work organization resulting from the implementation of AI?

- Increase in medical staff
- Increase in engineering staff
- Increase in computer science experts

- Need for spaces and equipment dedicated to data processing
- Increase in telemedicine activities
- Increase in smart working
- Other

Do you think that companies producing biomedical equipment used in your discipline will have to modify their production as a consequence of the implementation of AI?

- Yes
- No

If you answered yes to the previous question. Do you think that AI-integrated devices will be:

- More complex to use
- Easier to use
- More expensive
- Cheaper

What feeling does the topic of AI generate in you in relation to your future career?

- Concern
- Anxiety
- Enthusiasm
- Optimism
- Indifference
- Other

Section 4: ETHICAL IMPLICATIONS OF AI

Do you think it is right to entrust the treatment or diagnosis of a patient entirely to AI?

- Yes
- No

Do you think it is right for AI to analytically support medical personnel in treating or diagnosing a patient?

- Yes
- No

Do you think that the intervention of a medical figure is always necessary?

- Yes
- No

Do you think that in the event of medical-legal disputes, the doctor's professional responsibility is alleviated by the fact that he has used AI?

- Yes
- No

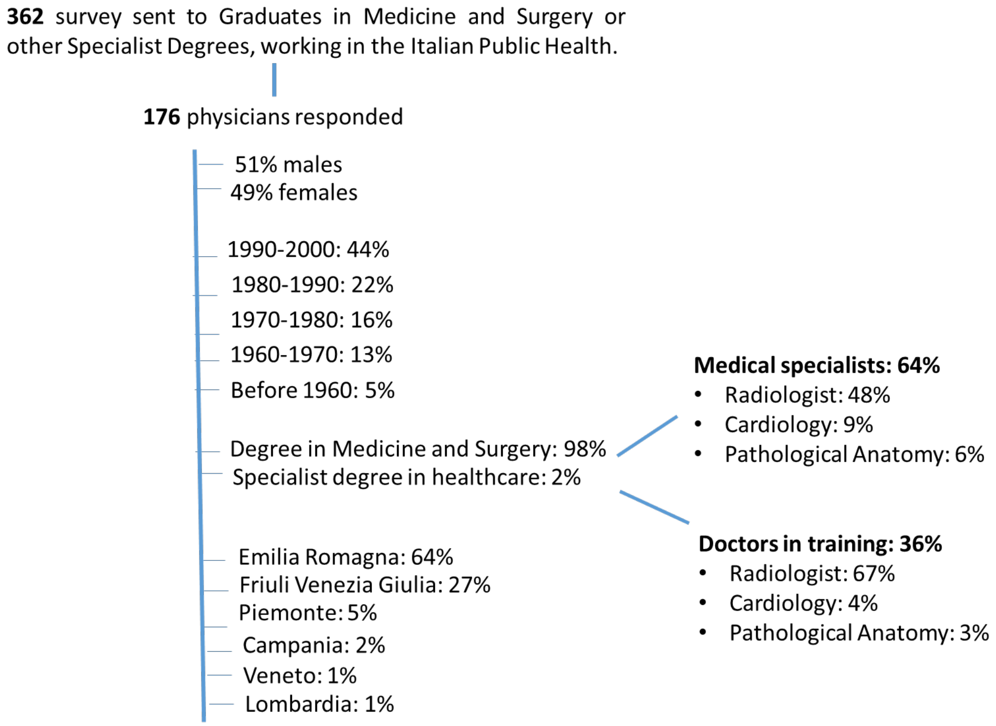

Fig 2: Graphical representation of the characteristics of the respondents.

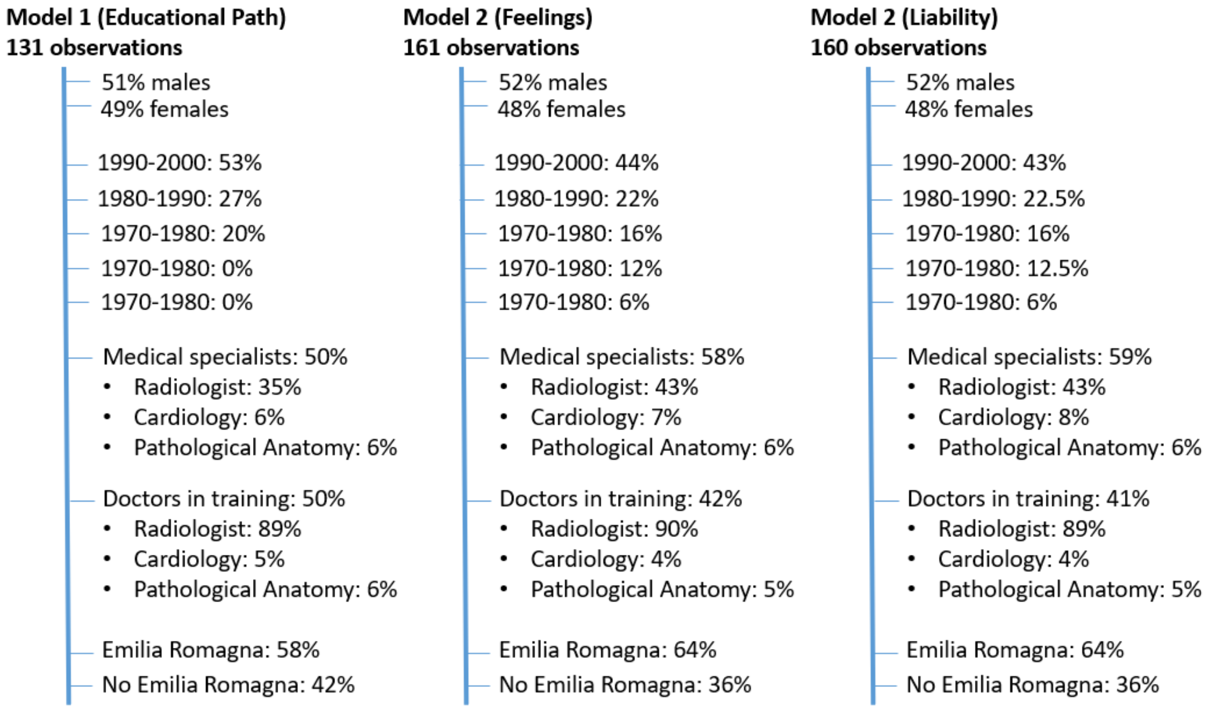

Fig 3: Graphical representation of the characteristics of the respondents in each model of Table 4.

Main Stata commands used for model estimation and diagnostic tests

```
* Model estimation for Educational path (used also for the variable Liability)
logit Educational i.Year i.Gender i.Noner i.Radiologist
* marginal effects
margins,dydx(*) post
* Odds ratios
logit Educational i.Year i.Gender i.Noner i.Radiologist, or
*Goodness-of-fit (Hosmer-Lemeshow test)
estat gof, group(10)

* Model estimation for Feelings
ologit Feelings i.Year i.Gender i.Noner i.Radiologist
* marginal effects
margins, dydx(*) post predict(outcome(1))
margins, dydx(*) post predict(outcome(2))
margins, dydx(*) post predict(outcome(3))
*Odds ratios
ologit Feelings i.Year i.Gender i.Noner i.Radiologist, or
*Brant test per Proportional odds check
brant
```

GVIF has been computed with the R command vif()

Fig 4: Codes for replicability
